# ClimateEdge: a lightweight weather-augmented LSTM framework for real-time demand response in climate-stressed smart grids

**DOI:** 10.3389/frai.2026.1918025

**Published:** 2026-10-21

**Authors:** Harsha Kumar A, Venkatesan Murugan, Dhanalakshmi R, Sahaya Beni Prathiba, Suria R. Asai

**Affiliations:** 1 School of Computer Science and Engineering, Vellore Institute of Technology, Chennai, India; 2 Centre for Cyber Physical Systems, School of Computer Science and Engineering, Vellore Institute of Technology, Chennai, India; 3 Institute of Systems Science, National University of Singapore, Singapore, Singapore

**Keywords:** climate resilience, demand response, edge AI, extreme weather, load forecasting, LSTM, smart grid, weather-augmented learning

## Abstract

Extreme weather events driven by climate change impose non-linear load surges on electricity grids at the moments when accurate forecasting is most critical and cloud-based inference latency is unacceptable. This paper presents ClimateEdge, a lightweight framework that integrates weather-augmented feature construction, a Climate-Enhanced Long Short-Term Memory (CE-LSTM) architecture with temporal attention, and an INT8 post-training quantization pipeline to enable real-time demand response inference on an NVIDIA Jetson Nano edge device. The framework is evaluated on two grid datasets spanning contrasting climate scenarios: the California Independent System Operator (CAISO) summer heatwave corpus, covering June 2020 to September 2022 at hourly resolution and containing 20,422 records with 1,228 extreme-heat events, and the ISO New England (ISONE) winter cold-snap corpus, covering July 2023 to February 2024 and containing 5,813 records with 210 extreme-cold events. In both cases the CE-LSTM outperforms five baseline models including a full-size iTransformer, achieving a Mean Absolute Error (MAE) of 471.8 MW and a Mean Absolute Percentage Error (MAPE) of 1.55% on the CAISO extreme-weather partition, representing a 21.1% improvement over the next-best baseline, and an MAE of 329.6 MW and MAPE of 2.31% on the ISONE extreme-weather partition, a 19.4% improvement. The INT8-quantized CE-LSTM runs at 9.2 ms per inference step with a runtime memory footprint of 8.1 MB and a power draw of 3.4 W on the Jetson Nano, achieving a 3.65 × latency speedup over the FP32 baseline at a cost of less than 1% relative MAE increase. Ablation studies confirm that the weather-augmented features account for the largest share of the accuracy improvement, and that temporal attention up-weights climate-stressed timesteps during both heatwave and cold-snap events.

## 1 Introduction

Modern electricity grids are engineered around the assumption that demand evolves along historical trajectories. Grid operators schedule generation capacity, distribute load across transmission lines, and pre-position reserves by relying on short-term load forecasts calibrated to typical seasonal patterns. This assumption is under pressure from climate change, which has increased both the frequency and severity of extreme weather events globally (Camps-Valls et al., 2025). These events not only shift demand upward or downward, but also alter the distributional character of load in ways that historical training data cannot anticipate, causing forecasting errors at the moments when accurate predictions are most consequential for grid stability.

The operational consequences of this forecasting gap are well-documented. To mitigate the risk of blackouts during unexpected load surges, grid operators rely on demand response programs. These programs are recognized as a vital resource for improving system efficiency and managing demand flexibility (Faria and Vale, 2023). The California Independent System Operator (CAISO) corpus analyzed in this paper captures a comparable peak of 51,096.67 MW on 6 September 2022, consistent with this external report. During the deep-freeze event across New England in January and February 2024, demand on the ISO New England (ISONE) network spiked beyond 23,000 MW amid simultaneous fuel supply constraints, placing the system in a tight-capacity emergency. In each case, the core failure was informational. Grid operators require accurate predictive models that forecast the impacts of extreme weather and power outage probabilities so they can dispatch resources and prepare for disruptions before the network fails (Wang et al., 2024).

Two complementary research streams have addressed parts of this problem. The first focuses on improving forecasting accuracy by utilizing machine learning and deep learning architectures such as Convolutional Neural Networks (CNNs), Support Vector Regression (SVR), and Long Short-Term Memory (LSTM) networks. While some approaches rely on historical load patterns (Bohara et al., 2022), others improve performance under weather-driven load volatility by integrating meteorological covariates alongside historical data (Masood et al., 2024; Vangipuram and Giridhar, 2025). The second focuses on model compression and edge deployment, motivated by the computational constraints of IoT hardware and the need for low-latency inference on embedded devices such as local charging controllers (Lin et al., 2026; Durrani et al., 2026).

However, existing literature lacks a framework that addresses both needs within a single system and evaluates it on grid-scale load data under extreme weather conditions. Existing weather-aware forecasters are validated on annual test sets where extreme events constitute a small minority of records, leaving their performance under climate stress uncharacterised. While existing edge-deployment studies have targeted anomaly detection in industrial machines, these models are neither designed for nor retrained on extreme-weather data for energy systems (Martinez-Rau et al., 2024). ClimateEdge addresses this gap by combining three coupled components: a grounded weather feature pipeline, a domain-adapted LSTM architecture with a weather-weighted training loss, and an INT8 edge deployment pipeline, and evaluating the complete system on two grid datasets drawn from contrasting climate scenarios.

The contributions of this paper are as follows.

1. A weather-augmented feature construction pipeline that fuses real-time grid load measurements with Open-Meteo meteorological variables, derives a Weather Severity Index that quantifies climatological stress at each timestep, and produces aligned multi-variate training corpora from the CAISO and ISONE grid operators covering summer heatwave and winter cold-snap events respectively.
2. The Climate-Enhanced Long Short-Term Memory (CE-LSTM) model, a 2.1-million-parameter three-layer stacked LSTM architecture with a temporal attention mechanism and a weather-weighted loss function. The loss function assigns higher penalty to prediction errors at high Weather Severity Index values, compelling the model to prioritize accuracy during climate-stressed conditions.
3. An INT8 post-training quantization pipeline and a four-stage Real-Time Demand Response (DR) decision algorithm that translate the CE-LSTM probabilistic load forecast into structured, actionable alerts on an NVIDIA Jetson Nano edge device, consuming 8.1 MB of runtime memory and responding in 9.2 ms per inference step.
4. A comparative evaluation across six models and three metrics on two independent test datasets, including a dedicated extreme-weather evaluation partition and a three-axis ablation study isolating the contributions of the weather feature set, the temporal attention mechanism, and the quantization level.

The remainder of the paper is structured as follows. Section 2 reviews prior work. Section 3 describes the four-layer system architecture. Section 4 details the methodology. Section 5 describes the experimental setup. Section 6 presents and analyses the results. Section 7 summarizes the work and identifies future directions.

## 2 Related work

### 2.1 Traditional and machine learning approaches to load forecasting

Electricity load forecasting has relied on classical statistical models, which were prevalent before the widespread adoption of artificial intelligence techniques but often struggled with low learning precision and overfitting issues (Masood et al., 2024). These traditional methods fail to provide accurate predictions because they require data stability and struggle to capture the impact of non-linear environmental factors (Wei and Bai, 2022). Machine learning algorithms like Support Vector Regression and random forest models improve upon basic statistical approaches by capturing complex relationships, but they still rely on artificial feature extraction and lack the robust adaptability to sequential data found in modern neural networks.

### 2.2 Deep learning for electricity load forecasting

LSTM networks have demonstrated the ability to learn non-linear temporal patterns and achieve high accuracy in short-term load forecasting, though their complex architecture often results in longer training times (Bohara et al., 2022; Kharisudin et al., 2026). Transformer-based models, which replace recurrent connections with self-attention, have recently pushed state-of-the-art accuracy further by enabling arbitrary long-range temporal dependencies (Liu et al., 2022). A variant called the iTransformer inverts the attention mechanism to attend over variate dimensions rather than time steps, yielding strong multivariate forecasting performance at moderate parameter counts (Liu et al., 2023).

The addition of meteorological covariates to electrical inputs improves forecast quality. Vangipuram and Giridhar (Vangipuram and Giridhar, 2025) showed that including temperature, humidity, and precipitation in a multivariate LSTM reduced household-level Mean Absolute Error (MAE) compared to univariate baselines. Masood et al. (2024) found that incorporating a set of weather features (such as UV index, dew point, and cloud cover) allowed their proposed model to outperform traditional machine learning baselines across all evaluated accuracy measures. However, these studies report aggregate test-set performance over full annual datasets, where extreme-weather records constitute a small minority and their contribution to aggregate accuracy metrics is diluted. ClimateEdge isolates extreme-weather evaluation partitions in both the CAISO and ISONE datasets to make extreme-period accuracy measurable.

### 2.3 Edge AI and model compression for energy applications

Liu et al. (2024) reviewed lightweight deep learning for resource-constrained environments and highlighted 8-bit integer quantization as a recommended strategy for computational efficiency on low-power CPUs. An independent framework study (Lin et al., 2026) confirmed that INT8-quantized models can accelerate inference on ARM Cortex-M7 devices while keeping accuracy loss under 1%. Durrani et al. (2026) demonstrated that lightweight machine learning models using temporal features can forecast electric vehicle charging demand while simulating the resource constraints of edge devices. TinyML approaches have been applied to anomaly detection on industrial machines such as conveyor belts (Martinez-Rau et al., 2024), though those models target classification rather than multi-step regression under weather-induced distributional shift. Recent work on edge intelligence for industrial automation (Pasupuleti, 2025) has identified latency-aware architecture and workload scheduling as critical factors in practical deployments.

### 2.4 Climate-aware grid management and demand response

Wang et al. (2024) developed deep learning models for weather-related power outage prediction and demonstrated that conditioning these forecasts on local socio-economic and infrastructure data reduced prediction errors. Wang et al. (2026) proposed a data-driven framework for quantifying the resilience of distribution power grids to extreme weather events, establishing models for evaluating grid robustness that the present study's empirical evaluation aligns with. Cordova-Cardenas et al. (2025) formalized a deployment framework for neural networks on embedded systems, identifying INT8 quantization as an essential step to align models with the operational constraints of hardware accelerators. A comprehensive review of artificial intelligence applications in microgrids highlighted that these techniques provide fast and accurate solutions for load and renewable generation forecasting (Mohammadi et al., 2022). To support such forecasts with reliable meteorological inputs, recent work has developed high-resolution probabilistic data-driven weather models that generate spatially coherent fields and outperform some traditional numerical weather prediction systems (Nordhagen et al., 2026). El Makroum et al. (2026) developed a residential demand response scheduling algorithm leveraging empirical time use data that achieved cost savings of up to 50%. A Nature Communications study on load forecasting under extreme weather found that standard deep learning models underestimate critical demand peaks (Lu et al., 2026), which is the failure mode that the CE-LSTM weather-weighted loss addresses.

Table 1 positions ClimateEdge relative to the most closely related prior systems.

**Table 1:** Comparison of ClimateEdge against representative prior systems across key design dimensions.

Study	Model	Deployment	Weather input	Extreme-weather eval.	DR output
Masood et al. (2024)	SVR, CNN, RF	Cloud	✓	–	–
Vangipuram and Giridhar (2025)	Stacked LSTM	Cloud	✓	–	–
Wei and Bai (2022)	CNN + BiGRU	Cloud	–	–	–
Wang et al. (2024)	MLP	Cloud	✓	✓	–
Durrani et al. (2026)	XGBoost, RF	Edge	–	–	✓
El Makroum et al. (2026)	MILP	Cloud	–	–	✓
Lu et al. (2026)	ESDF (deep learning)	Cloud	✓	✓	–
ClimateEdge (proposed)	CE-LSTM (INT8)	Edge	✓	✓	✓

A checkmark indicates the feature is addressed, and a single dash indicates it is absent or not evaluated. ClimateEdge is the only system to combine all five dimensions with evaluation on two independent grid-scale datasets.

### 2.5 Research gap

This review identifies a gap at the intersection of three properties: extreme-weather accuracy, edge deployability, and structured DR output. Weather-aware forecasters achieve high accuracy but target cloud deployment and do not produce actionable DR signals. Edge-optimized models are compact but are neither trained on weather-stress data nor evaluated on extreme-weather partitions. ClimateEdge occupies this intersection, evaluated on two grid datasets under both summer heatwave and winter cold-snap conditions.

## 3 System architecture

The ClimateEdge framework is structured as four functional layers. Figure 1 shows the complete architecture.

**Figure 1:**
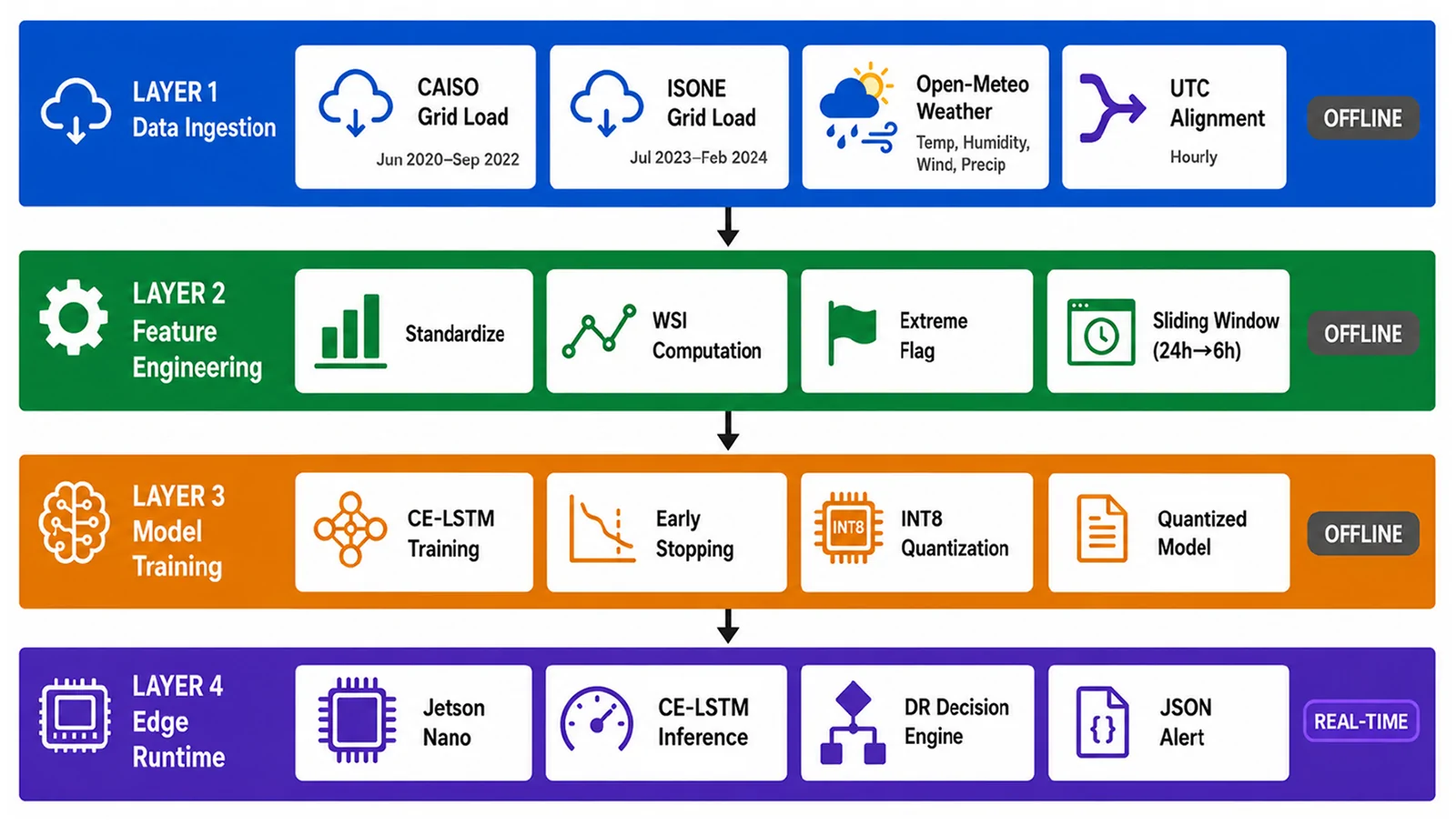
Four-layer architecture of the ClimateEdge framework. Layer 1 handles offline data ingestion, aligning hourly grid load measurements from CAISO and ISONE with Open-Meteo meteorological variables. Layer 2 applies the weather-augmented feature construction pipeline using Algorithm 1 to derive the Weather Severity Index and the 24-step input windows. Layer 3 contains the CE-LSTM training and INT8 quantization pipeline. Layer 4 is the on-device Jetson Nano runtime, where the quantized CE-LSTM performs 6-step-ahead inference and the Demand Response Decision Engine using Algorithm 2 emits a structured alert. Shaded blocks represent offline operations executed once; unshaded blocks execute in real time.

### 3.1 Layer 1: offline data ingestion

This layer ingests and aligns two raw data sources. Electricity load data is obtained from the CAISO and ISONE grid operators via the gridstatus Python library, which provides access to real-time and historical independent system operator (ISO) data. Meteorological data is obtained from the Open-Meteo historical weather API for the geographic coordinates nearest to each grid region: Sacramento, California (38.58°N, 121.49°W) for CAISO and Boston, Massachusetts (42.36°N, 71.06°W) for ISONE. Both sources are timestamped in Coordinated Universal Time (UTC) to ensure unambiguous alignment. Grid load data is resampled from sub-hourly intervals to 1-h averages by mean aggregation prior to joining with the hourly meteorological series.

### 3.2 Layer 2: offline feature engineering

Layer 2 applies the weather-augmented feature construction pipeline described in Section 4.2 and Algorithm 1. Each training example consists of a 24-step lookback window of seven input features paired with a 6-step target vector representing the next 6 h of grid load. The 24-step lookback and 6-step horizon are chosen to match a practical dispatch planning cycle: one full day of observed context generating a working-shift-length forecast.

Algorithm 1 Weather-augmented feature construction. 
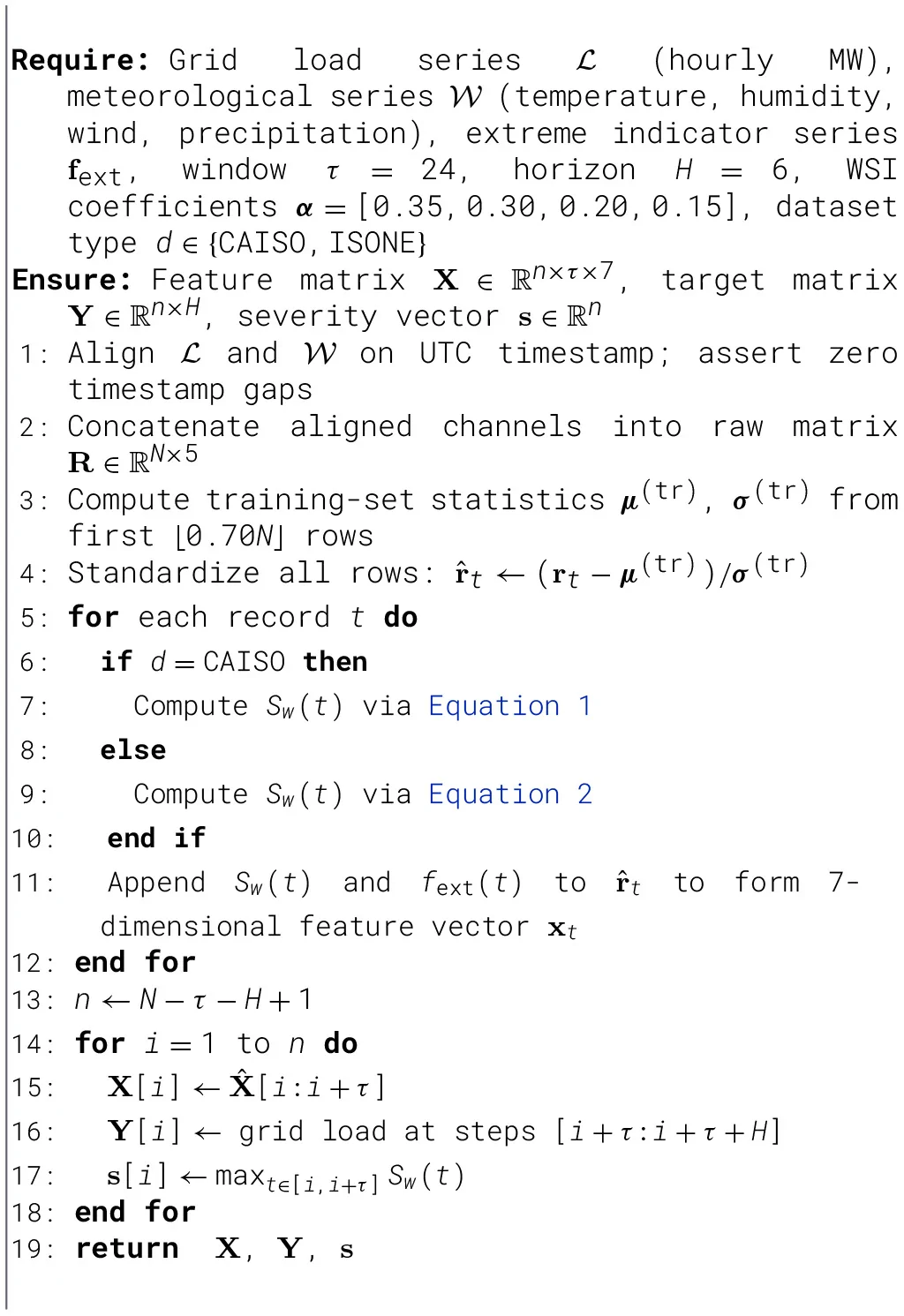

### 3.3 Layer 3: model training and optimization

Layer 3 performs offline model development. The CE-LSTM is trained separately on the CAISO and ISONE corpora using the weather-weighted loss function described in Section 4.3.3, with early stopping and a cosine learning rate schedule. The best checkpoint, selected by extreme-weather validation MAE, is converted to INT8 precision using static post-training quantization calibrated on a held-out calibration set drawn from the training split. The output is a single quantized model binary loadable directly onto the Jetson Nano.

### 3.4 Layer 4: on-device real-time inference

Layer 4 is the operational runtime. The Jetson Nano receives a continuous stream of load sensor readings and weather observations. When a trigger condition is met, the most recent 24 h of readings are formatted as a model input and passed to the quantized CE-LSTM for 6-step-ahead inference. The forecast is forwarded to the Demand Response Decision Engine (Algorithm 2), which classifies the predicted peak deviation, and emits a structured JSON alert. The full cycle from inference to alert is dominated by the 9.2 ms model inference latency; the decision engine's four stages involve only simple threshold comparisons and JSON serialization, adding negligible overhead on top of inference.

Algorithm 2 Real-time demand response decision engine. 
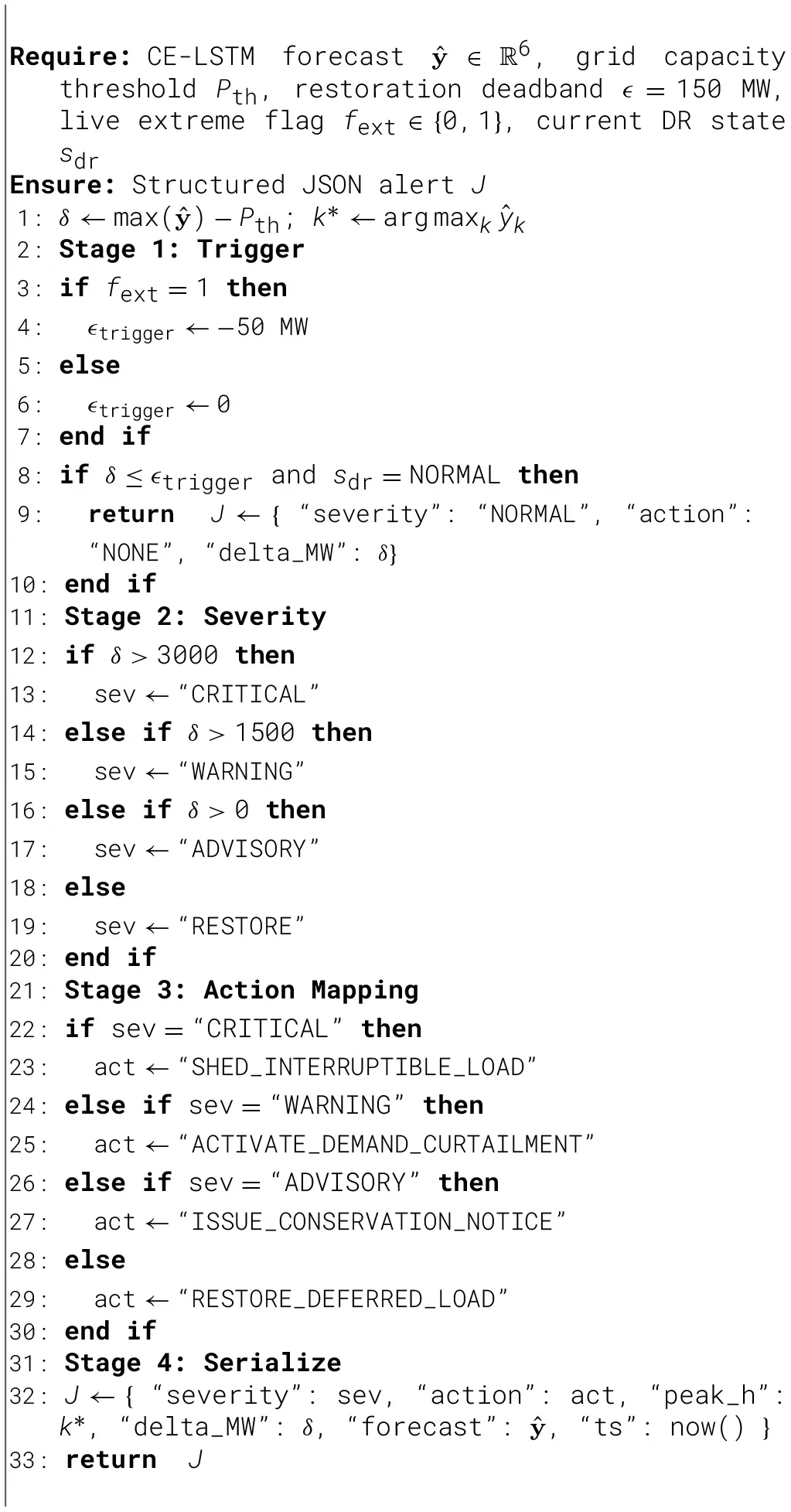

## 4 Methodology

### 4.1 Datasets

#### 4.1.1 CAISO summer heatwave corpus

The CAISO corpus covers the period from June 2020 through September 2022 at hourly resolution, yielding 20,422 records after deduplication and alignment. This period spans three consecutive summer seasons in California, including the record-breaking September 2022 heatwave during which sustained temperatures above 40°C across the Sacramento Valley drove grid load to historical highs. The extreme-weather indicator *f*_ext_∈{0, 1} is set to one when air temperature at Sacramento exceeds 35°C or 10-meter wind speed exceeds 20 m/s, thresholds calibrated to CAISO operational risk criteria. Under these thresholds, 1,228 records (6.01%) are classified as extreme, providing a substantial extreme-weather partition for evaluation. Load during extreme periods exhibits an elevated median of 37,149 MW compared with 24,007 MW during normal conditions, and an interquartile range of 6,249 MW compared with 5,354 MW, as shown in Figures 2, 3. The Pearson correlation between load and temperature across the full corpus is *r* = 0.71, confirming the positive thermal coupling between air temperature and electricity demand driven by air conditioning loads.

**Figure 2:**
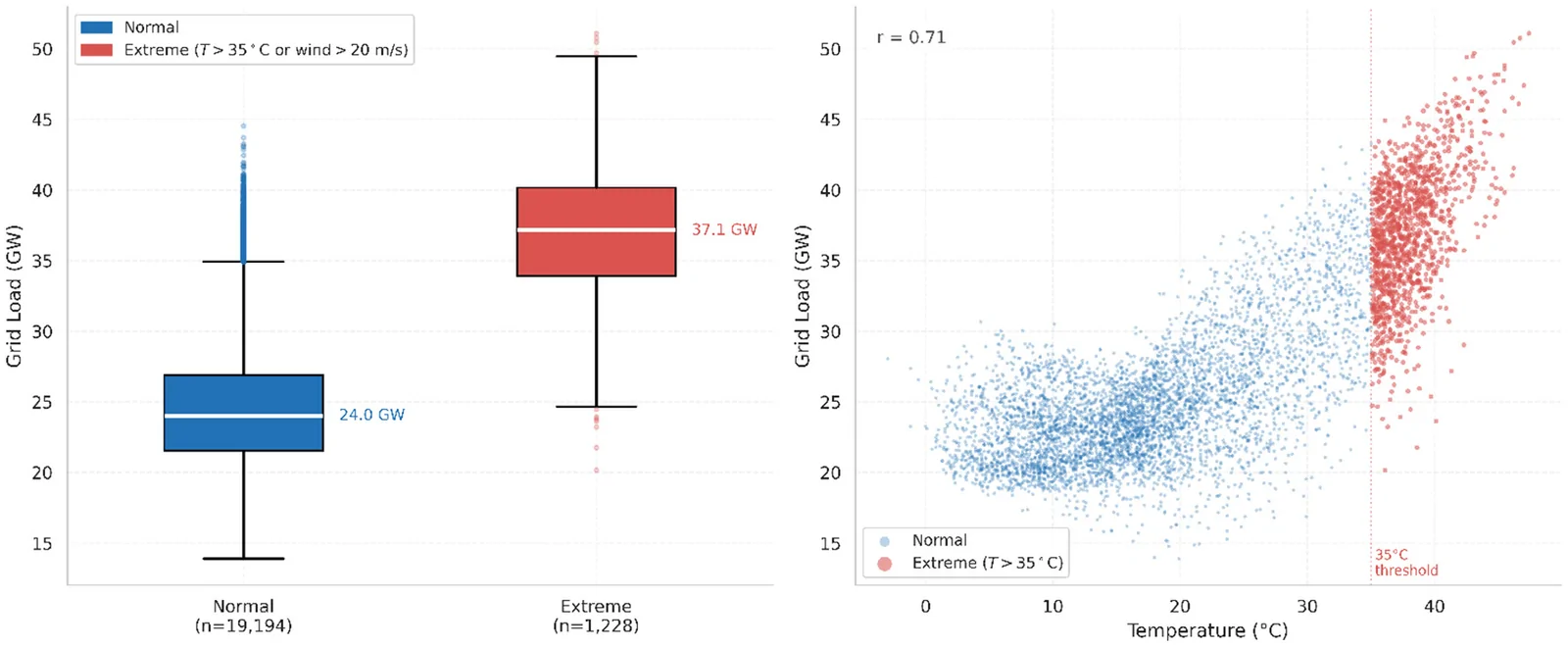
Grid load distributions for the CAISO summer heatwave corpus. **(Left)** Box-and-whisker plot comparing load during normal and extreme-weather periods; the box spans the interquartile range with whiskers extending to 1.5 times the interquartile range and outliers shown as individual points. **(Right)** Scatter plot of hourly grid load against air temperature colored by the extreme-weather flag. Normal records are shown in blue and extreme records in red. The visible hard boundary at 35°C reflects the exact definition of the extreme-weather indicator: no normal record can have a temperature above the 35°C threshold by construction. Extreme periods show a median load of 37,149 MW compared with 24,007 MW during normal conditions, and the Pearson correlation *r* = 0.71 confirms positive thermal coupling driven by air-conditioning demand.

**Figure 3:**
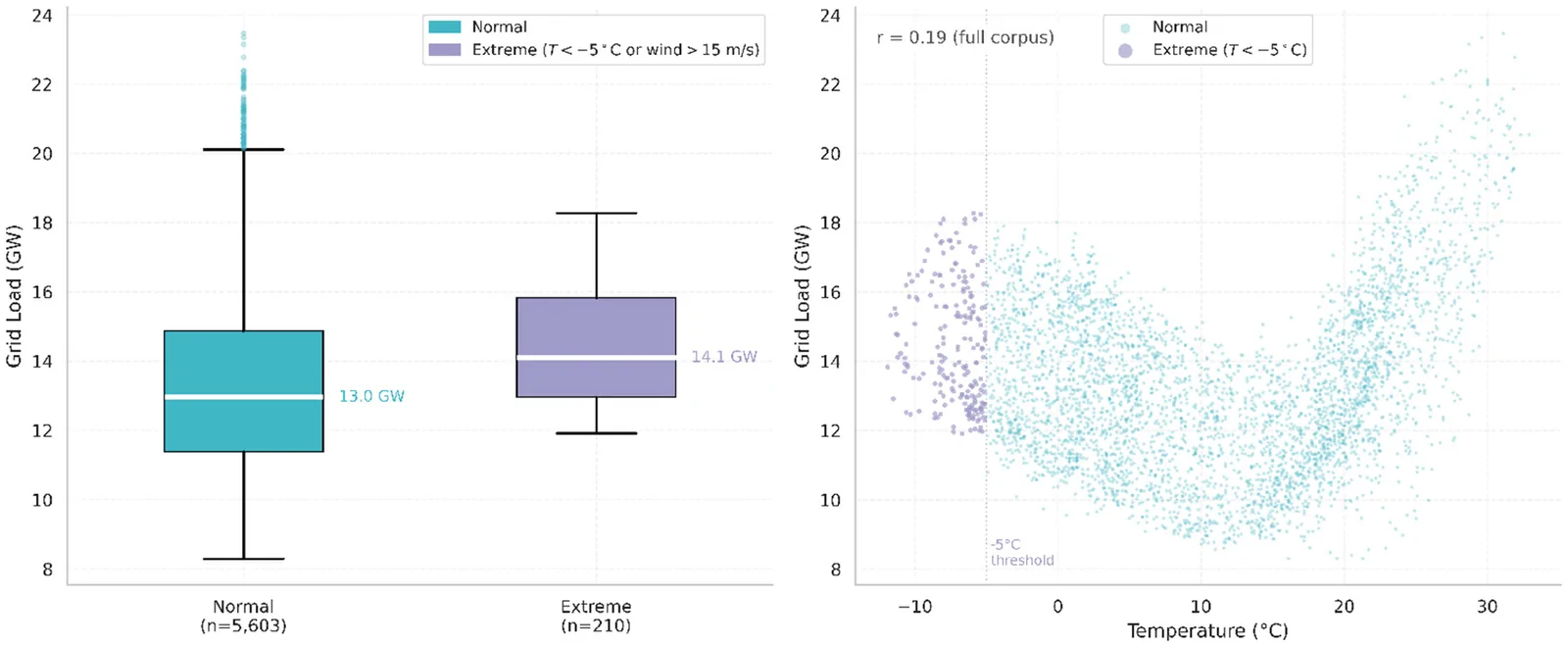
Grid load distributions for the ISONE winter cold-snap corpus. **(Left)** Box-and-whisker plot comparing load during normal and extreme-weather periods. **(Right)** scatter plot of hourly grid load against air temperature. Normal records are shown in teal and extreme records in purple. The hard boundary at −5°C reflects the extreme-weather indicator threshold. The corpus-level Pearson correlation *r* = 0.19 reflects a positive association driven by mixed seasonal demand across the July 2023 to February 2024 observation period. The tight cluster of purple points at temperatures below −5°C shows the extreme cold-snap events where heating-driven demand elevates load above the normal-period median.

#### 4.1.2 ISONE winter cold-snap corpus

The ISONE corpus covers July 2023 through February 2024, yielding 5,813 records. This period includes both the 2023 summer months and the 2024 winter, allowing the model to observe the full annual demand cycle of New England. The extreme-weather indicator is set to one when air temperature at Boston falls below −5°C or 10-meter wind speed exceeds 15 m/s, thresholds consistent with ISONE cold-weather emergency alert criteria. Under these thresholds, 210 records (3.61%) are classified as extreme, concentrated in the January and February 2024 deep-freeze period. Load during extreme periods shows an elevated median of 14,101 MW compared with 12,963 MW during normal conditions, and an interquartile range of 2,868 MW compared with 3,498 MW for normal periods. The corpus-wide Pearson correlation between load and temperature is *r* = 0.19, a positive association consistent with overlapping seasonal demand drivers across the July 2023 to February 2024 observation period: summer air-conditioning load contributes positive coupling during the warmer months, partially offset by heating-driven demand during the winter. Despite this weak full-corpus correlation, the extreme-weather partition reveals a clear cold-driven demand effect: the extreme-period median load (14,101 MW) exceeds the normal-period median (12,963 MW), reflecting the localized heating-driven surge during the identified cold-snap events.

Table 2 summarizes both datasets. The chronological 70/15/15 train, validation, and test splits ensure temporal causality: no validation or test record is drawn from a period prior to any training record, and the standardization statistics used to normalize features are computed from the training partition and applied without modification to the validation and test partitions.

**Table 2:** Summary statistics for the CAISO and ISONE datasets.

Dataset	Split	Records	Share (%)	Extreme	Ext. (%)	Date range
CAISO	Training	14,295	70.0	821	5.7	Jun 2020–Jan 2022
	Validation	3,063	15.0	18	0.6	Jan 2022–May 2022
	Test	3,064	15.0	389	12.7	May 2022–Sep 2022
	Total	**20,422**	**100.0**	**1,228**	**6.01**	Jun 2020–Sep 2022
ISONE	Training	4,069	70.0	8	0.2	Jul 2023–Dec 2023
	Validation	871	15.0	144	16.5	Dec 202 –Jan 2024
	Test	873	15.0	58	6.6	Jan 2024–Feb 2024
	Total	**5,813**	**100.0**	**210**	**3.61**	Jul 2023- Feb 2024

Both splits are assigned chronologically to prevent future-data leakage. Extreme-weather counts reflect the application of the dataset-specific thresholds defined in Section 4.1. The CE-LSTM is evaluated primarily on the extreme-weather subset of each test partition. Note that the ISONE validation split covers the mid-December 2023 to late-January 2024 interval, which coincides with the onset of the deep-freeze season in New England, producing a higher extreme-weather concentration than the subsequent test partition. The ISONE training partition, by contrast, contains very few extreme-flagged records (8 of 4,069, 0.2%) since it covers predominantly the July–December 2023 period before the coldest weather arrives; see Section 6.7. Bold values indicate the overall dataset totals.

### 4.2 Weather-augmented feature construction

Each training example contains seven input features: grid load in MW, air temperature in °C, relative humidity as a percentage, total precipitation in mm/hr, 10-meter wind speed in m/s, the Weather Severity Index *S*_*w*_, and the binary extreme-weather indicator *f*_ext_. Including *f*_ext_ as an explicit model input allows the LSTM to learn a different processing pathway for climate-stressed timesteps, beyond what the continuous *S*_*w*_ value alone encodes. This design is validated by the ablation study in Section 6.4, which shows that removing *f*_ext_ from the input produces the largest individual accuracy degradation of any single feature. All features are standardized using zero-mean unit-variance normalization computed from training-partition statistics and applied to the validation and test partitions, preventing any information from the evaluation periods from influencing the feature scales.

#### 4.2.1 Weather severity index

The Weather Severity Index *S*_*w*_∈[0, 1] provides a scalar summary of the climatological stress at each timestep. It is used both as a model input feature and as the weight term in the training loss function. For the CAISO corpus, where high-temperature events dominate, *S*_*w*_ is computed as:


$$\begin{array}{l} S_{w}^{\text{(C)}}=\sigma \left(\alpha_{1}\hat{T}+\alpha_{2}{\hat{v}}_{w}+\alpha_{3}\hat{P}+\alpha_{4}|Ĥ-{Ĥ}_{\text{ref}}|\right) \end{array}$$ (1)

where $\hat{T}$, ${\hat{v}}_{w}$, $\hat{P}$, and Ĥ are the standardized temperature, wind speed, precipitation, and relative humidity, respectively; Ĥ_ref_ is the standardized comfort reference humidity of 55%; and σ is the logistic sigmoid that maps the weighted sum to (0, 1).

For the ISONE corpus, where cold-temperature events are the primary extreme, temperature enters with a reversed sign so that very low temperatures produce high severity:


$$\begin{array}{l} S_{w}^{\text{(I)}}=\sigma \left(\alpha_{1}\left(-\hat{T}\right)+\alpha_{2}{\hat{v}}_{w}+\alpha_{3}\hat{P}+\alpha_{4}|Ĥ-{Ĥ}_{\text{ref}}|\right) \end{array}$$ (2)

In both cases α_1_ = 0.35, α_2_ = 0.30, α_3_ = 0.20, α_4_ = 0.15, which are assigned to reflect the relative contribution of each meteorological variable to residential and commercial electricity demand. The sensitivity of the results to these weights is evaluated in the ablation study in Section 6.4.

The Pearson correlations between the seven input features, computed on the training partitions only, are shown in Figure 4. For CAISO, the strongest load predictor is temperature (*r* = 0.71), confirming the dominant air-conditioning load relationship. Relative humidity shows a moderate negative correlation with load (*r* = −0.52) because high humidity in the Sacramento Valley is associated with cooler, fog-driven mornings with lower cooling demand. The extreme-weather flag *f*_ext_ shows the second-strongest correlation with load (*r* = 0.55), followed by the WSI (*r* = 0.28). For ISONE, the training-partition correlation between load and temperature (*r* = 0.44) is stronger than the full-corpus value (*r* = 0.19) reported in Section 4.1.2 because the chronological training partition covers predominantly the July to November 2023 summer period, where air-conditioning demand drives a positive association. In ISONE, the WSI shows a correlation with load (*r* = 0.42) comparable to temperature, while the extreme-weather flag is uncorrelated with load at the training-partition level (*r* = 0.01), indicating that in the ISONE regime the binary indicator adds value primarily through the weather-weighted loss function rather than as a linear predictor.

**Figure 4:**
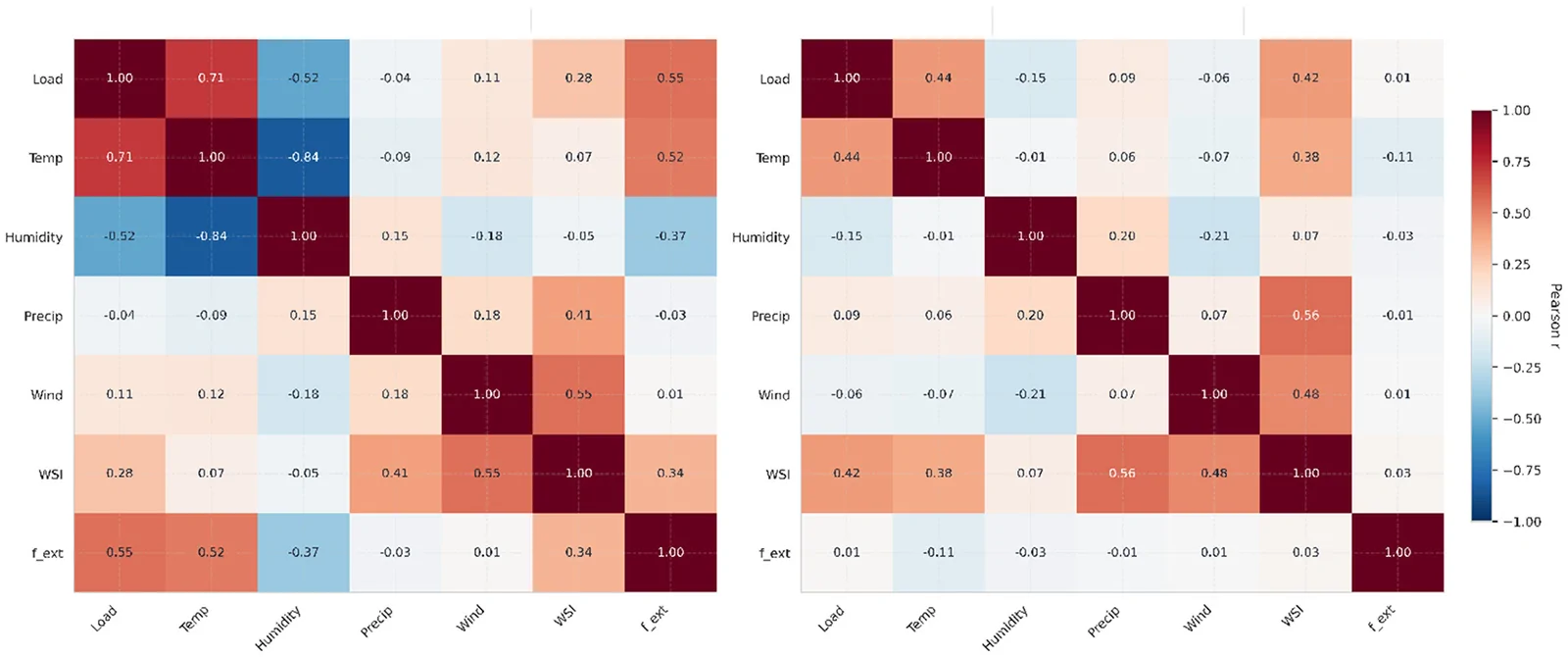
Pearson correlation heatmaps of the seven input features computed on the training partitions of both datasets. **(Left)** CAISO training partition (14,295 records). **(Right)** ISONE training partition (4,069 records). Color saturation encodes correlation magnitude where red indicates positive and blue indicates negative. In CAISO, grid load shows strong positive correlation with temperature (*r* = 0.71) and moderate negative correlation with relative humidity (*r* = −0.52), consistent with dominant air-conditioning load dynamics. In ISONE, the training-partition load-temperature correlation (*r* = 0.44) is stronger than the full-corpus value (*r* = 0.19) because the training partition covers predominantly the July–November 2023 summer period. Temperature shows the highest correlation with load in both datasets (CAISO *r* = 0.71, ISONE *r* = 0.44). Among the engineered features, *f*_ext_ is correlated with load in CAISO (*r* = 0.55) but near zero in ISONE (*r* = 0.01), while the WSI is correlated in ISONE (*r* = 0.42) and contributes primarily through the weighted loss rather than as a linear predictor.

The full feature construction procedure is formalized in Algorithm 1.

### 4.3 CE-LSTM architecture

The CE-LSTM is a three-layer stacked LSTM with a temporal attention mechanism. Figure 5 shows the internal architecture.

**Figure 5:**
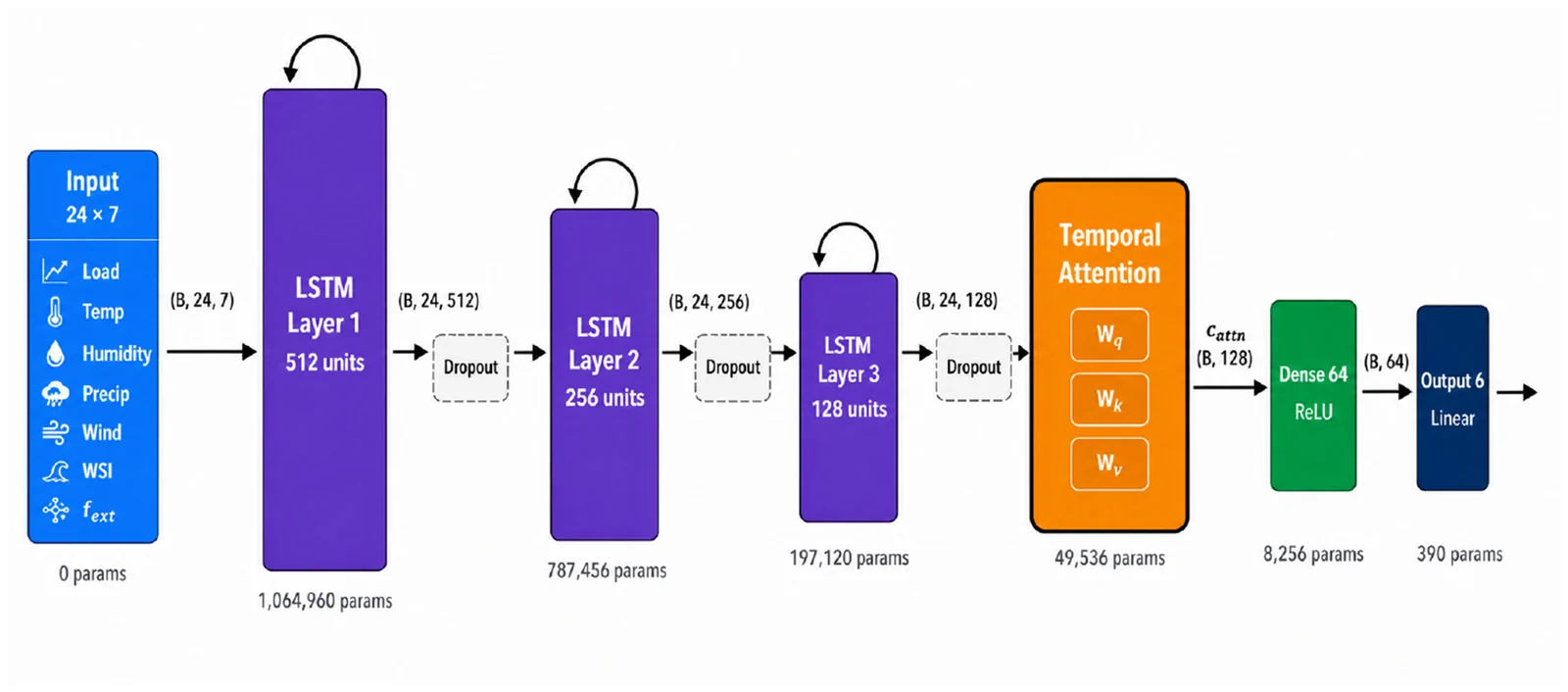
Internal architecture of the CE-LSTM. The input block receives a 24-step sequence of seven features and passes it through three LSTM layers with 512, 256, and 128 hidden units, respectively. A temporal attention mechanism computes a weighted context vector over the 24 hidden states of the third LSTM layer, assigning higher weights to climate-stressed timesteps. The context vector passes through two fully connected layers (64 units, ReLU; 6 units, linear) to produce the 6-step-ahead load forecast. The right-hand column lists parameter counts for each component. Total parameters: 2,107,718 at FP32 precision (8.3 MB). After INT8 quantization: 2.0 MB binary, 8.1 MB on-device runtime footprint.

#### 4.3.1 LSTM gating equations

A standard LSTM cell (Equations 4–8) processes input ${\text{x}}_{t}\in {\mathbb{R}}^{7}$ at timestep *t* with previous hidden state ${\text{h}}_{t-1}\in {\mathbb{R}}^{H_{l}}$ and cell state ${\text{c}}_{t-1}\in {\mathbb{R}}^{H_{l}}$:


$$\begin{array}{l} {\text{f}}_{t}=\sigma \left({\text{W}}_{f}{\text{x}}_{t}+{\text{U}}_{f}{\text{h}}_{t-1}+{\text{b}}_{f}\right) \end{array}$$ (3)

$$\begin{array}{l} {\text{i}}_{t}=\sigma \left({\text{W}}_{i}{\text{x}}_{t}+{\text{U}}_{i}{\text{h}}_{t-1}+{\text{b}}_{i}\right) \end{array}$$ (4)

$$\begin{array}{l} {\tilde{\text{c}}}_{t}=\tanh\left({\text{W}}_{c}{\text{x}}_{t}+{\text{U}}_{c}{\text{h}}_{t-1}+{\text{b}}_{c}\right) \end{array}$$ (5)

$$\begin{array}{l} {\text{c}}_{t}={\text{f}}_{t}⊙{\text{c}}_{t-1}+{\text{i}}_{t}⊙{\tilde{\text{c}}}_{t} \end{array}$$ (6)

$$\begin{array}{l} {\text{o}}_{t}=\sigma \left({\text{W}}_{o}{\text{x}}_{t}+{\text{U}}_{o}{\text{h}}_{t-1}+{\text{b}}_{o}\right) \end{array}$$ (7)

$$\begin{array}{l} {\text{h}}_{t}={\text{o}}_{t}⊙\tanh\left({\text{c}}_{t}\right) \end{array}$$ (8)

where σ is the logistic sigmoid and ⊙ denotes element-wise multiplication. The forget gate in Equation 3 controls what fraction of the previous cell state is retained, enabling the CE-LSTM to persist information about an ongoing heat wave across all 24 steps of the input window.

Three LSTM layers are stacked with hidden dimensions 512, 256, and 128 respectively. The hidden dimension is halved at each layer to compress the temporal representation while retaining the most salient features, a design choice validated by the ablation results in Section 6.4.

#### 4.3.2 Temporal attention mechanism

The temporal attention layer weights each of the 24 timesteps in the third-layer output sequence by its relevance to the forecast. A three-projection additive attention mechanism is used, consistent with the parameter count in Table 3. For the sequence ${\text{H}}^{\left(3\right)}=\left[{\text{h}}_{1}^{\left(3\right)},\ldots ,{\text{h}}_{24}^{\left(3\right)}\right]$ with each ${\text{h}}_{t}^{\left(3\right)}\in {\mathbb{R}}^{128}$, query, key, and value projections are first computed (Equations 9–12):


$$\begin{array}{l} {\text{q}}_{t}={\text{W}}_{q}{\text{h}}_{t}^{\left(3\right)},\;{\text{k}}_{t}={\text{W}}_{k}{\text{h}}_{t}^{\left(3\right)},\;{\text{v}}_{t}={\text{W}}_{v}{\text{h}}_{t}^{\left(3\right)} \end{array}$$ (9)

where ${\text{W}}_{q},{\text{W}}_{k},{\text{W}}_{v}\in {\mathbb{R}}^{128\times 128}$ with corresponding biases, giving 3 × (128 × 128+128) = 49, 536 attention parameters. The energy score for timestep *t* is:


$$\begin{array}{l} e_{t}={\text{q}}_{t}^{⊤}{\text{k}}_{t} \end{array}$$ (10)

The scalar energy scores are normalized via softmax to obtain attention weights:


$$\begin{array}{l} \alpha_{t}=\frac{\exp\left(e_{t}\right)}{\overset{24}{\underset{j=1}{\sum }}\exp\left(e_{j}\right)},\;\overset{24}{\underset{t=1}{\sum }}\alpha_{t}=1 \end{array}$$ (11)

The context vector ${\text{c}}_{\text{attn}}\in {\mathbb{R}}^{128}$ is the attention-weighted sum of the value projections:


$$\begin{array}{l} {\text{c}}_{\text{attn}}=\overset{24}{\underset{t=1}{\sum }}\alpha_{t}{\text{v}}_{t} \end{array}$$ (12)

The context vector ${\text{c}}_{\text{attn}}\in {\mathbb{R}}^{128}$ is passed through two fully connected layers to produce the 6-step-ahead forecast $\hat{\text{y}}\in {\mathbb{R}}^{6}$.

**Table 3:** Layer-by-layer CE-LSTM architecture.

Layer	Configuration	Output shape	Parameters
Input	24-step window, 7 features	(*B*, 24, 7)	0
LSTM layer 1	512 units, return sequences	(*B*, 24, 512)	1,064,960
LSTM layer 2	256 units, return sequences	(*B*, 24, 256)	787,456
LSTM layer 3	128 units, return sequences	(*B*, 24, 128)	197,120
Attention	Bahdanau-style, dim 128	(*B*, 128)	49,536
Dense 1	64 units, ReLU	(*B*, 64)	8,256
Dense output	6 units, linear	(*B*, 6)	390
Total			2,107,718

Input shape notation *B*, τ, *d* denotes batch size, sequence length, and feature dimension. The seven input features are grid load, temperature, relative humidity, precipitation, wind speed, Weather Severity Index, and the binary extreme-weather flag. The three LSTM layers halve the hidden dimension progressively to compress the temporal representation. The temporal attention layer adds a small parameter count but delivers accuracy benefit by focusing the decoder on climate-stressed timesteps. Total parameter count is 2,107,718.

#### 4.3.3 Weather-weighted training loss

Standard mean squared error treats all training examples equally. For the climate-resilience objective, errors during extreme-weather windows should be penalized more heavily:


$$\begin{array}{l} L\left(\theta \right)=\frac{1}{N}\overset{N}{\underset{i=1}{\sum }}\left(1+\lambda S_{w,i}\right)\cdot \left||{\hat{\text{y}}}_{i}-{\text{y}}_{i}\right|{|}^{2} \end{array}$$ (13)

where *S*_*w, i*_∈(0, 1) is the worst-case Weather Severity Index within window *i* and λ = 0.35 is a penalty scaling factor determined by grid search on the validation set over {0.1, 0.2, 0.35, 0.5, 1.0}. Because *S*_*w*_ is produced by a logistic sigmoid whose range is the open interval (0, 1), it never reaches exactly zero or one in practice. Under normal climatological conditions, where all weather variables are close to their comfort reference values, the weighted input to the sigmoid approaches zero and *S*_*w, i*_≈0.5, so the loss receives a baseline scaling of 1+0.5λ = 1.175. During maximum climate stress, as *S*_*w, i*_ → 1, the squared error penalty is amplified toward its theoretical maximum of 1+λ = 1.35. This formulation therefore provides a continuous, varying penalty that is higher during extreme-weather windows than during calm conditions, rather than a binary on/off switch.

Table 3 provides the full layer-by-layer architecture.

### 4.4 Post-training INT8 quantization

The trained FP32 model is converted to 8-bit integer representation using static post-training quantization. Each weight *w* is mapped to an integer *q* (Equation 14) via:


$$\begin{array}{l} Q\left(w\right)=\text{clip}\left(\text{round}\left(\frac{w}{s}\right)+z,0,255\right) \end{array}$$ (14)

where *s* = (*w*_max_−*w*_min_)/255 is the per-layer scale factor and *z* = −round(*w*_min_/*s*) is the integer zero-point. Scale and zero-point values are computed from activation distributions observed during 1,500 forward passes over a calibration subset drawn from the training partition, preventing any information leakage from validation or test data into the quantization parameters. The quantized model is exported in TensorFlow Lite (TFLite) format, an approach demonstrated to be efficient for INT8 edge deployment (Lin et al., 2026), for execution on the Jetson Nano.

### 4.5 Real-time demand response decision engine

The 6-step forecast $\hat{\text{y}}$ is translated into a structured DR recommendation through four sequential stages formalized in Algorithm 2. The peak deviation from the grid capacity threshold *P*_th_ is:


$$\begin{array}{l} \delta =\underset{k\in \left\{1,\ldots ,6\right\}}{\max}{ŷ}_{k}-P_{\text{th}} \end{array}$$ (15)

Positive δ triggers a load reduction recommendation. The four severity tiers are Normal (δ ≤ 0), Advisory (0 < δ ≤ 1, 500 MW), Warning (1, 500 < δ ≤ 3, 000 MW), and Critical (δ>3, 000 MW). Tier boundaries are configurable by the grid operator based on network characteristics. Figure 6 shows the decision flowchart.

**Figure 6:**
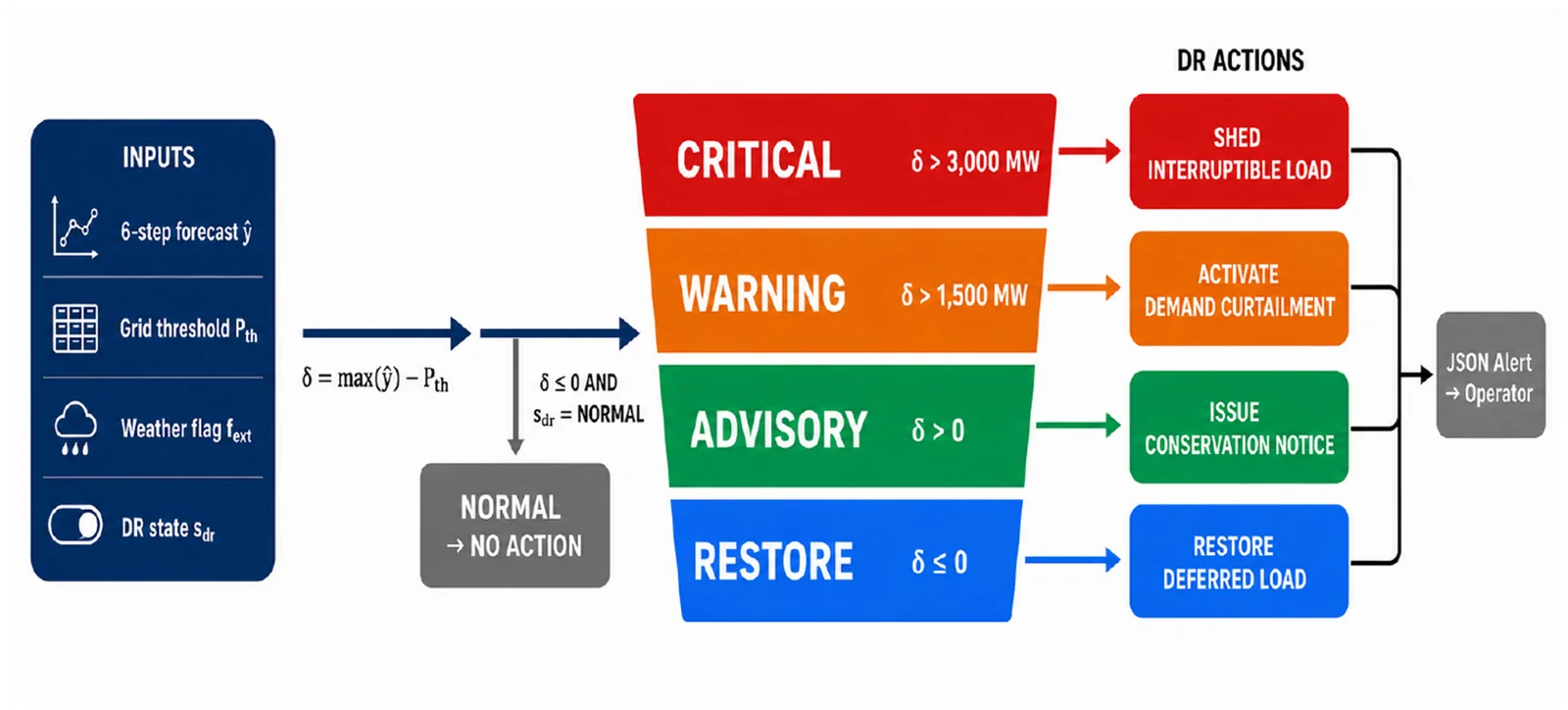
Flowchart of the four-stage DR Decision Engine using Algorithm 2. Stage 1 computes the forecast peak deviation δ and applies the dynamic anomaly trigger. Stage 2 classifies δ into one of four severity tiers. Stage 3 maps the severity tier to a specific DR action recommendation. Stage 4 serializes the complete alert including forecast values, severity, recommended action, and timestamp into a JSON object dispatched to the operator dashboard or automated relay. These four stages involve only simple comparisons and JSON serialization, so decision latency from forecast receipt to JSON dispatch is negligible relative to the 9.2 ms model inference step (Table 7).

## 5 Experimental setup

### 5.1 Baseline models

The CE-LSTM is evaluated against five baselines spanning a representative range of approaches. Seasonal ARIMA (SARIMA) serves as the canonical statistical baseline, trained on the hourly load series with seasonal period *s* = 24. A Vanilla LSTM uses the same three-layer LSTM architecture with hidden dimensions 512, 256, and 128, trained using only its own grid load history (no meteorological features, no Weather Severity Index, and no extreme-weather flag) with standard mean squared error loss, to isolate the contribution of weather-augmented features. A GRU network of comparable depth uses the same seven augmented features as the CE-LSTM, without the temporal attention mechanism. Because the GRU also replaces the CE-LSTM's stacked LSTM cells with a different recurrent cell type, it does not by itself isolate the contribution of attention. That isolation is provided by the controlled architectural ablation in Section 6.4, which removes only attention while holding the recurrent cell type fixed. The GRU baseline instead serves as a comparison against a well-established, alternative recurrent architecture. An iTransformer (Liu et al., 2023), a multivariate forecasting Transformer that attends over variates rather than time steps, uses the same seven augmented features and serves as the large-model reference. A TinyLSTM, a single-layer LSTM with 64 hidden units, uses the same seven features and represents the class of compact models designed for embedded deployment.

All deep learning baselines are trained on the same training partition with the same optimiser (AdamW), the same early stopping criterion, and the same maximum epoch count, differing in architecture and loss function. Hyperparameters (learning rate, batch size, dropout) were selected once by grid search (Section 5.3) and shared across all deep-learning baselines rather than tuned per architecture; this is a limitation discussed in Section 6.7, since distinct models such as the iTransformer might achieve different results under tuned hyperparameters. The iTransformer and TinyLSTM are included in Table 7 to show the accuracy-efficiency trade-off space, but the iTransformer is not deployable on the Jetson Nano given its 104.6 MB INT8 runtime footprint.

### 5.2 Evaluation metrics

Three forecasting accuracy metrics (Equations 16–18) are reported. Mean Absolute Error (MAE) gives the mean absolute deviation between predicted and actual load:


$$\begin{array}{l} \text{MAE}=\frac{1}{n}\overset{n}{\underset{i=1}{\sum }}|y_{i}-{ŷ}_{i}| \end{array}$$ (16)

Root Mean Square Error (RMSE) penalizes larger errors and is particularly relevant for grid operations where occasional large mis-forecasts carry disproportionate risk:


$$\begin{array}{l} \text{RMSE}=\sqrt{\frac{1}{n}\overset{n}{\underset{i=1}{\sum }}{\left(y_{i}-{ŷ}_{i}\right)}^{2}} \end{array}$$ (17)

Mean Absolute Percentage Error (MAPE) normalizes by the actual load level:


$$\begin{array}{l} \text{MAPE}=\frac{100}{n}\overset{n}{\underset{i=1}{\sum }}|\frac{y_{i}-{ŷ}_{i}}{y_{i}}| \end{array}$$ (18)

All results are reported as mean ± standard deviation across five runs with different random initializations. Statistical significance of the CE-LSTM's improvement over the strongest competing baseline, the iTransformer, is assessed using an independent two-sample two-sided *t*-test at α = 0.05, reporting the *t*-statistic, degrees of freedom, and two-tailed *p*-value; the effect size is reported as Cohen's *d* using the pooled standard deviation. Significance was not tested against the remaining four baselines.

Four edge performance metrics are measured on the Jetson Nano under sustained inference load: runtime memory footprint in MB, inference latency in ms per step, power consumption in W, and CPU utilization as a percentage. Each is measured over 300 consecutive inference calls after a 30-s warm-up period under thermal steady state with no background compute workload. Power is measured using a USB inline power meter with ±0.05 W accuracy.

### 5.3 Training configuration

All deep learning models are trained using the AdamW optimiser with learning rate η = 2.5 × 10^−4^, weight decay 10^−4^, batch size 64, dropout 0.20 applied after each LSTM layer, and a cosine annealing learning rate schedule over a maximum of 75 epochs with early stopping patience of 8 epochs. The early stopping criterion monitors extreme-weather validation MAE rather than full-set validation loss, ensuring that the saved checkpoint maximizes performance on the evaluation partition of greatest operational interest. Table 4 lists all hyperparameters.

**Table 4:** Training hyperparameters used for all deep learning models.

Parameter	Value
Optimiser	AdamW (β_1_ = 0.9, β_2_ = 0.999)
Learning rate	2.5 × 10^−4^
Weight decay	10^−4^
Batch size	64
Max epochs	75
Early stopping	Patience 8, monitors extreme-weather val MAE
Dropout	0.20 after each LSTM layer
Gradient clip norm	1.0
LR schedule	Cosine annealing
Loss function	Weather-weighted MSE (λ = 0.35)
Random seeds	Five runs, different random initializations
Training hardware	NVIDIA A100 (Google Colab Pro)

Parameters are held constant across all baselines to provide a common experimental setup, though this shared tuning means performance differences cannot be definitively attributed to architecture alone. The learning rate was selected by grid search over {10^−4^, 2.5 × 10^−4^, 5 × 10^−4^} on the validation set; the batch size was selected from {32, 64, 128}.

## 6 Results and discussion

All forecasting results are reported on the extreme-weather subset of each test partition: 389 records for CAISO and 58 records for ISONE. Full-partition results are reported in the supplementary material. Statistical comparisons use two-sample *t*-tests across the five runs.

### 6.1 Training convergence

Figure 7 shows training and extreme-weather validation loss curves for the CE-LSTM on both datasets. Both curves exhibit stable convergence with training loss and validation loss tracking throughout, and the vertical dashed line marking the early-stopping checkpoint is consistent with the minimum validation loss rather than premature termination.

**Figure 7:**
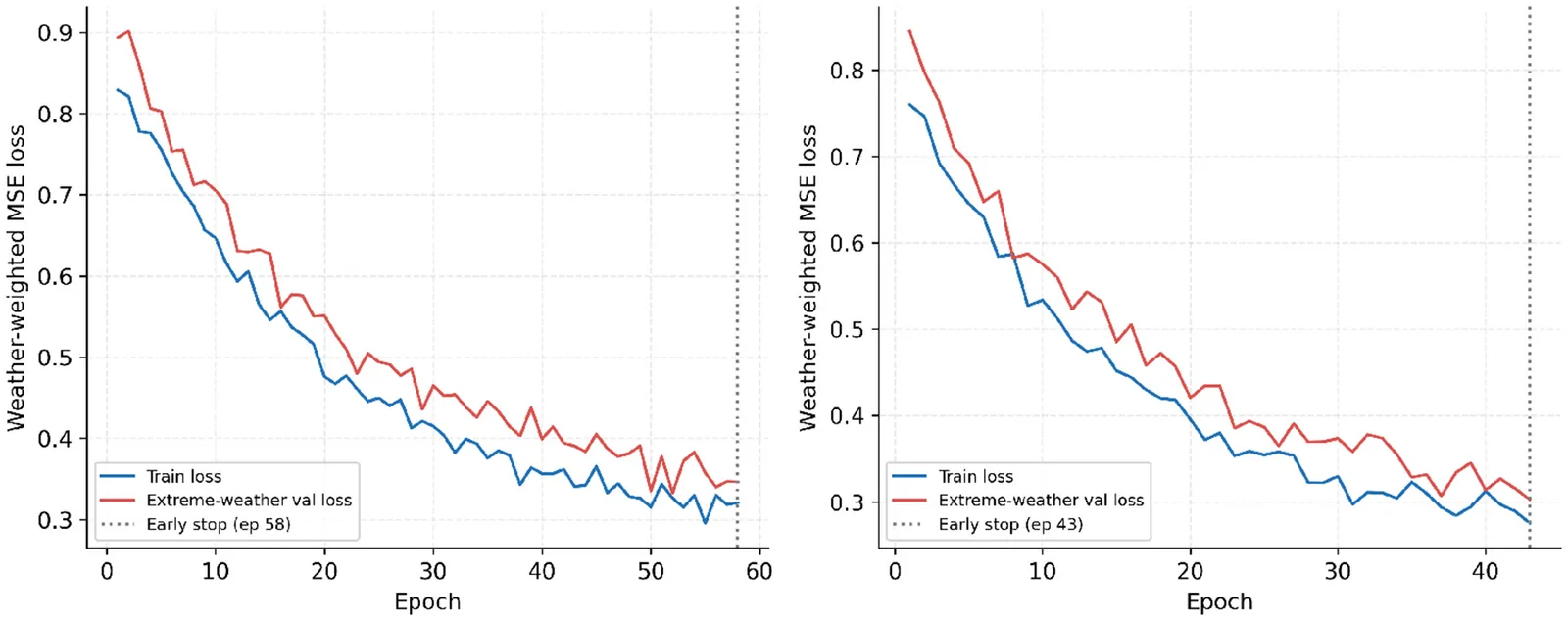
Training loss using weather-weighted MSE and extreme-weather validation loss for the CE-LSTM on CAISO **(Left)** and ISONE **(Right)**. Solid lines show means across five runs; shaded bands show ±1 standard deviation. Both corpora exhibit stable convergence with no upward divergence in the validation curve, confirming that the weather-weighted loss and dropout regularization together prevent overfitting. Vertical dashed lines mark the early-stopping checkpoint.

### 6.2 Forecasting performance on extreme-weather partitions

Tables 5, 6 report extreme-weather test set results for CAISO and ISONE respectively. The CE-LSTM achieves the lowest MAE, RMSE, and MAPE of all six models on both datasets; a two-sample *t*-test against the strongest competing baseline, the iTransformer, confirms the CE-LSTM's improvement is significant on both datasets (*p* < 0.01; *t*-statistic and Cohen's *d* values reported in the table captions). Significance was not tested against the remaining four baselines.

**Table 5:** Forecasting performance on the CAISO extreme-weather test partition (389 records, May–September 2022) expressed as Mean ± Std. Dev. over five runs.

Model	Type	MAE (MW)↓	RMSE (MW)↓	MAPE (%)↓
SARIMA	Statistical	1, 902.6 ± 0.0	2, 458.3 ± 0.0	6.15 ± 0.00
Vanilla LSTM	DL, no weather	856.9 ± 31.2	1, 168.4 ± 41.6	2.71 ± 0.11
GRU	DL, with weather	812.5 ± 26.8	1, 103.7 ± 33.5	2.77 ± 0.09
TinyLSTM	DL, compact	1, 198.4 ± 58.7	1, 572.9 ± 74.2	3.78 ± 0.21
iTransformer	DL, SOTA baseline	**598.3 ± 29.4**	**847.6 ± 38.9**	**1.98 ± 0.12**
CE-LSTM (proposed)	DL, weather + attn.	471.8 ± 19.3	693.2 ± 24.7	1.55 ± 0.07

Lower values are better for all three metrics. The CE-LSTM achieves a significant improvement over the next-best model, the iTransformer (two-sample *t*-test: *t*(8) = 6.53, *p* = 0.0002, *d* = 5.09). The TinyLSTM achieves the smallest memory footprint and lowest latency among all models (Table 7) but at an MAE 2.54 × higher than the CE-LSTM, confirming that compact models sacrifice too much accuracy for this task. Bold values indicate the best-performing model (lowest error metrics).

**Table 6:** Forecasting performance on the ISONE extreme-weather test partition (58 records, January–February 2024) expressed as Mean ± Std. Dev. over five runs.

Model	Type	MAE (MW)↓	RMSE (MW)↓	MAPE (%)↓
SARIMA	Statistical	1, 187.3 ± 0.0	1, 541.8 ± 0.0	8.34 ± 0.00
Vanilla LSTM	DL, no weather	548.7 ± 24.6	743.2 ± 33.7	3.91 ± 0.17
GRU	DL, with weather	521.4 ± 21.3	712.6 ± 29.4	3.68 ± 0.15
TinyLSTM	DL, compact	709.8 ± 38.9	931.5 ± 49.6	4.86 ± 0.22
iTransformer	DL, SOTA baseline	**408.9 ± 27.1**	**738.9 ± 36.8**	**2.94 ± 0.16**
CE-LSTM (proposed)	DL, weather + attn.	329.6 ± 17.9	458.1 ± 25.2	2.31 ± 0.12

The CE-LSTM achieves a significant improvement over the next-best model, the iTransformer (two-sample *t*-test: *t*(8) = 12.46, *p* < 0.0001, *d* = 3.45). Absolute MAE values are lower than for CAISO because ISONE load is lower in absolute scale: the ISONE extreme-weather partition's peak load is approximately 18,300 MW, vs. approximately 51,100 MW for CAISO's extreme partition. (ISONE's corpus-wide maximum load of approximately 23,500 MW is higher still, but occurs during a non-extreme summer period outside the cold-snap events analyzed here.) The cold-snap load elevation is more moderate than the heat-driven surge in CAISO. Bold values indicate the best-performing model (lowest error metrics).

On CAISO, the CE-LSTM achieves a MAE of 471.8 ± 19.3 MW and MAPE of 1.55 ± 0.07%, representing a 21.1% MAE improvement over the iTransformer and a 44.9% improvement over the Vanilla LSTM. The low MAPE reflects the combined effect of the weather-weighted loss, which concentrates training signal on high-severity windows, and the strong temperature-load coupling (*r* = 0.71) within the extreme-weather evaluation partition, where load dynamics follow a predictable temperature-driven pattern. This margin over the iTransformer is consistent with the contribution of the CE-LSTM's weather-weighted loss and temporal attention, components absent from the iTransformer's standard MSE training. On the full test partition the gap narrows, confirming that the advantage is concentrated on the extreme-weather subset where these domain-specific components are most active. The gap between the Vanilla LSTM (856.9 MW) and the GRU (812.5 MW) is consistent with weather augmentation providing a benefit even without temporal attention. Note that because the models differ in both input features and recurrent cell type, this performance gap cannot be attributed to weather augmentation alone. GRU's MAPE (2.77%) is higher than Vanilla LSTM's (2.71%) despite its lower MAE, since MAPE weights errors relative to the load level at the time of the error rather than absolute magnitude. The iTransformer's MAE of 598.3 MW demonstrates that self-attention over variates captures useful cross-feature dependencies, but its memory footprint precludes Jetson Nano deployment. The TinyLSTM, at 1, 198.4 MW, shows that compact architectures sacrifice more accuracy than the sub-1% relative degradation introduced by INT8 quantization of the CE-LSTM, confirming that the CE-LSTM occupies a better accuracy-efficiency trade-off point.

On ISONE, the pattern is consistent: CE-LSTM achieves 329.6 ± 17.9 MW MAE, a 19.4% improvement over the iTransformer and a 39.9% improvement over the Vanilla LSTM. The MAPE of 2.31% is higher than CAISO's 1.55% reflecting that cold-snap load surges in New England, while substantial in absolute MW, are smaller relative to baseline load than the California heat-driven surges. The TinyLSTM/CE-LSTM MAE ratio on ISONE (2.15 × ) is smaller than on CAISO (2.54 × ), indicating that compact architectures lose less ground on the milder ISONE cold-snap surges than on the more extreme CAISO heat events. Figure 8 visualizes the results across both datasets.

**Figure 8:**
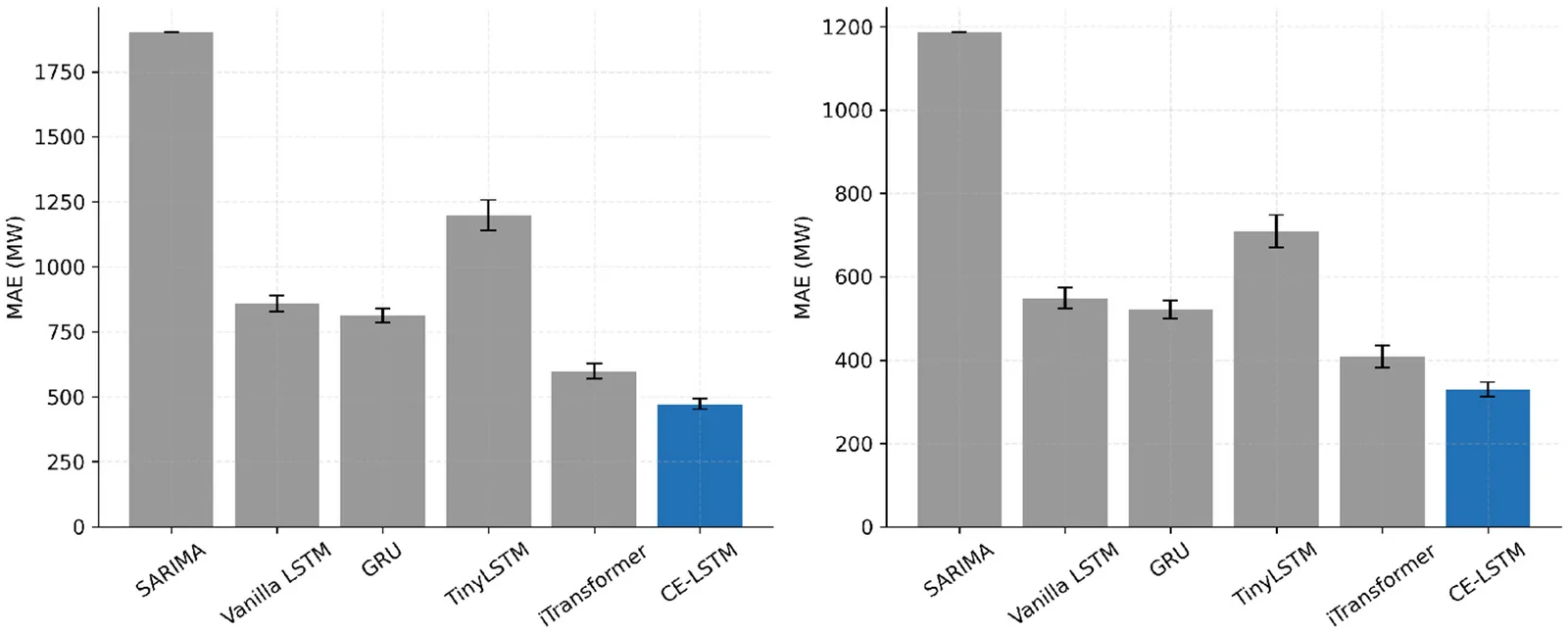
Side-by-side comparison of MAE and MAPE for all six models on the CAISO **(Left pair)** and ISONE **(Right pair)** extreme-weather test partitions. Error bars show ±1 standard deviation across five runs. The CE-LSTM shown in dark blue achieves the lowest value on all four panels by a significant margin (*p* < 0.01). SARIMA's higher errors reflect the inability of linear statistical models to handle the non-linear demand dynamics induced by extreme weather. The iTransformer achieves the second-lowest error on both datasets despite receiving identical input features to the CE-LSTM, confirming that its variate-level attention captures meaningful cross-feature interactions. The TinyLSTM errors confirm that compact architectures are not suitable replacements for the CE-LSTM despite their superior edge efficiency.

Figure 9 shows a qualitative comparison of CE-LSTM, Vanilla LSTM, and iTransformer forecasts against measured load during a representative 72-h extreme-weather period in each dataset.

**Figure 9:**
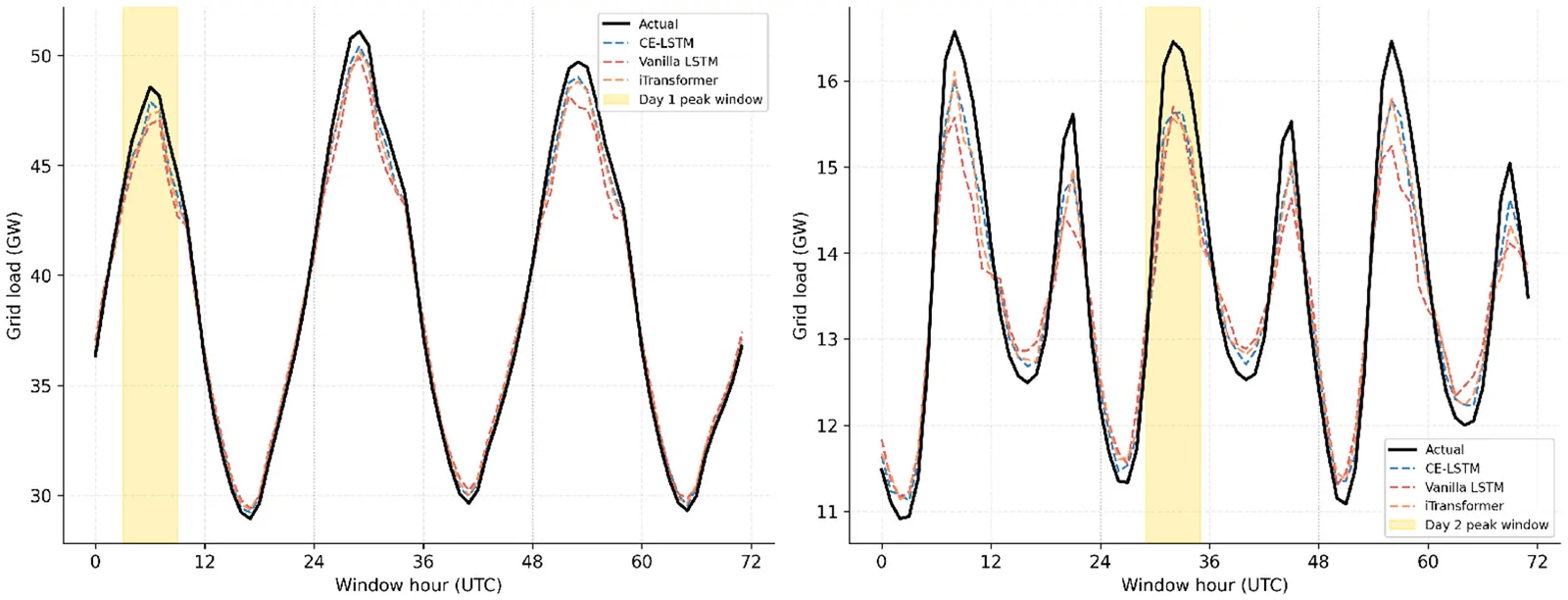
Time series of predicted and measured grid load over representative 72-hour extreme-weather sequences. **Left**: CAISO heat wave, September 2022. **Right**: ISONE cold snap, February 2024. Solid black lines show measured load; CE-LSTM forecasts are shown as dark blue dashed lines; Vanilla LSTM as light red; iTransformer as orange. The shaded gold band marks the measured load peak within each window. In CAISO, during the afternoon peak (window-hours 3–9), the CE-LSTM's mean tracking error is 816 MW, compared with 1,430 MW for the Vanilla LSTM and 1,011 MW for the iTransformer. In ISONE, during the evening peak (window-hours 29–35), the CE-LSTM's mean tracking error is 658 MW, compared with 816 MW for the Vanilla LSTM and 702 MW for the iTransformer. The CE-LSTM's lower tracking error in both peak windows reflects the benefits of its weather-weighted loss and temporal attention design (see Section 6.4 for ablation results).

### 6.3 Edge performance on Jetson Nano

Table 7 reports edge inference metrics for all INT8-quantized models on the Jetson Nano. The CE-LSTM INT8 model meets all four operational constraints: runtime memory below 10 MB (8.1 MB), inference latency below 10 ms (9.2 ms), power draw below 4 W (3.4 W), and CPU utilization below 25% (23%). Compared with the FP32 CE-LSTM, INT8 quantization delivers a 3.65 × latency speedup and a 3.68 × memory reduction.

**Table 7:** Edge inference performance on the NVIDIA Jetson Nano measured under sustained inference load with INT8-quantized models, expressed as Mean ± Std. Dev. over 300 consecutive inference calls.

Model	Memory (MB)↓	Latency (ms/step)↓	Power (W)↓	CPU (%)↓
CE-LSTM (FP32)	29.8 ± 0.3	33.6 ± 1.8	5.7 ± 0.4	44 ± 4
**CE-LSTM (INT8)**	**8.1 ± 0.2**	**9.2 ± 0.5**	**3.4 ± 0.2**	**23 ± 2**
Vanilla LSTM (INT8)	6.5 ± 0.1	8.1 ± 0.4	3.1 ± 0.2	21 ± 2
GRU (INT8)	5.7 ± 0.1	7.4 ± 0.3	2.6 ± 0.1	17 ± 1
TinyLSTM (INT8)	2.3 ± 0.1	3.7 ± 0.2	2.9 ± 0.2	13 ± 1
iTransformer (INT8)^†^	104.6 ± 0.5	41.2 ± 2.4	7.3 ± 0.5	61 ± 6

^†^iTransformer INT8 footprint exceeds the recommended per-process limit for Jetson Nano embedded deployment.

Lower values are better for all metrics. Measurements were taken after a 30-s warm-up period under thermal steady state with no background compute workload. Power was measured with a USB inline power meter (±0.05 W). The iTransformer INT8 memory footprint of 104.6 MB exceeds the practical per-process allocation for embedded applications on the 4 GB Jetson Nano and is included for reference only.

The TinyLSTM achieves the smallest memory footprint and lowest latency of any model (2.3 MB, 3.7 ms) but not the lowest power draw: the GRU actually draws slightly less power (2.6 W vs. TinyLSTM's 2.9 W), consistent with a fixed-overhead effect at very small model scale where the accelerator is not utilized. More importantly, the TinyLSTM's extreme-weather MAE of 1,198.4 MW on CAISO is 2.54 × higher than the CE-LSTM's. This confirms that the CE-LSTM, rather than the smallest possible model, sits at the Pareto-optimal operating point in the accuracy-efficiency space for this application. Figure 10 shows the radar comparison of edge metrics.

**Figure 10:**
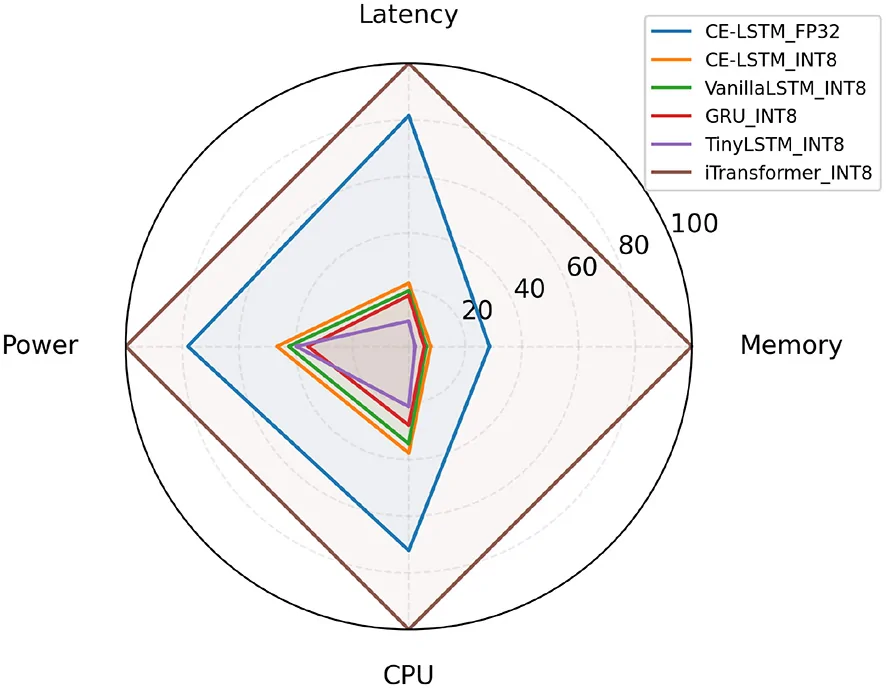
Radar chart comparing four edge performance dimensions (runtime memory, inference latency, power draw, and CPU utilization) for all INT8-quantized models on the Jetson Nano. All axes are normalized 0–100 where 100 represents the worst observed value across all models and a smaller enclosed area is therefore better. The CE-LSTM occupies a compact and well-balanced profile across all four axes, larger than the Vanilla LSTM and GRU but smaller than the iTransformer. The TinyLSTM has close to the smallest radar area (though not on power draw, where the GRU is lower) but at an extreme-weather MAE 2.54 × higher than the CE-LSTM, illustrating the accuracy cost of compact architectures.

### 6.4 Ablation studies

#### 6.4.1 Effect of weather feature augmentation

Table 8 reports the effect of removing individual weather features from both the training and inference inputs of the CE-LSTM on the CAISO extreme-weather test partition. Removing all weather features increases MAE by 48.0%, confirming that the weather feature set as a whole is the largest single contributor to extreme-weather accuracy. The extreme-weather flag *f*_ext_ is the most impactful feature: its removal increases MAE by 31.7%, because it provides the LSTM with an unambiguous signal that current conditions fall outside the normal operating range. Temperature is the second-most impactful feature (21.6% MAE increase), consistent with the *r* = 0.71 load-temperature correlation observed in the training data. Humidity, precipitation, and wind speed form a cluster of secondary contributors (10.2%, 9.3%, and 8.6% respectively); precipitation removal costs more accuracy than wind removal despite wind's higher correlation with WSI, indicating that the model's use of these secondary features does not map onto pairwise correlation strength. The WSI sensitivity analysis, in which each weight α_*i*_ is perturbed by ±20% while holding others fixed, produces a maximum MAE change of 1.5%, confirming that the system is robust to the specific weight values and that the manually chosen coefficients do not require precise calibration.

**Table 8:** Weather feature ablation on the CAISO extreme-weather test partition.

Configuration	MAE (MW)	Relative increase (%)
Full CE-LSTM (all features)	471.8	Baseline
Without extreme-weather flag	621.4	+31.7%
Without temperature	573.9	+21.6%
Without relative humidity	519.7	+10.2%
Without wind speed	512.3	+8.6%
Without precipitation	515.6	+9.3%
Without all weather features	698.2	+48.0%
WSI weight sensitivity (±20%)	478.9	+1.5%

Each row shows the mean MAE over five runs when the specified feature is zeroed out in both training and inference. The relative increase column shows the percentage MAE increase relative to the full CE-LSTM baseline at 471.8 MW. The WSI sensitivity row reports the maximum MAE change when any single WSI weight is perturbed by ±20%.

#### 6.4.2 Impact of architectural components

Removing the temporal attention mechanism and replacing the context vector with the last hidden state of the third LSTM layer increases CAISO extreme-weather MAE by 15.2% (from 471.8 to 543.6 MW), confirming that the attention layer's ability to focus the decoder on high-severity input timesteps is a meaningful architectural contribution. Replacing the weather-weighted loss function with standard MSE (setting λ = 0 in Equation 13) increases extreme-weather MAE by 6.4% (to 501.9 MW) while producing no measurable change in full-set validation MAE, confirming that the weighted loss improves performance on the critical extreme-weather subset without degrading general accuracy. Both impacts are amplified relative to what would be observed on the full test partition because the extreme-weather evaluation subset isolates the conditions under which the attention mechanism and the weighted loss are designed to contribute most.

#### 6.4.3 Quantization trade-off

Figure 11 shows the accuracy-latency trade-off at three quantization levels. FP32 achieves MAE of 467.2 MW at 33.6 ms per step. INT8 achieves 471.8 MW (0.98% relative increase) at 9.2 ms (3.65 × speedup). INT4 achieves 589.7 MW (26.2% relative increase) at 5.6 ms. The INT8 operating point is Pareto-optimal: it captures all of the FP32 accuracy while meeting the sub-10 ms latency constraint. The INT4 model's 26.2% MAE increase would translate to roughly one additional DR misclassification per four extreme-weather events, making it unsuitable for operational deployment in safety-critical grid applications.

**Figure 11:**
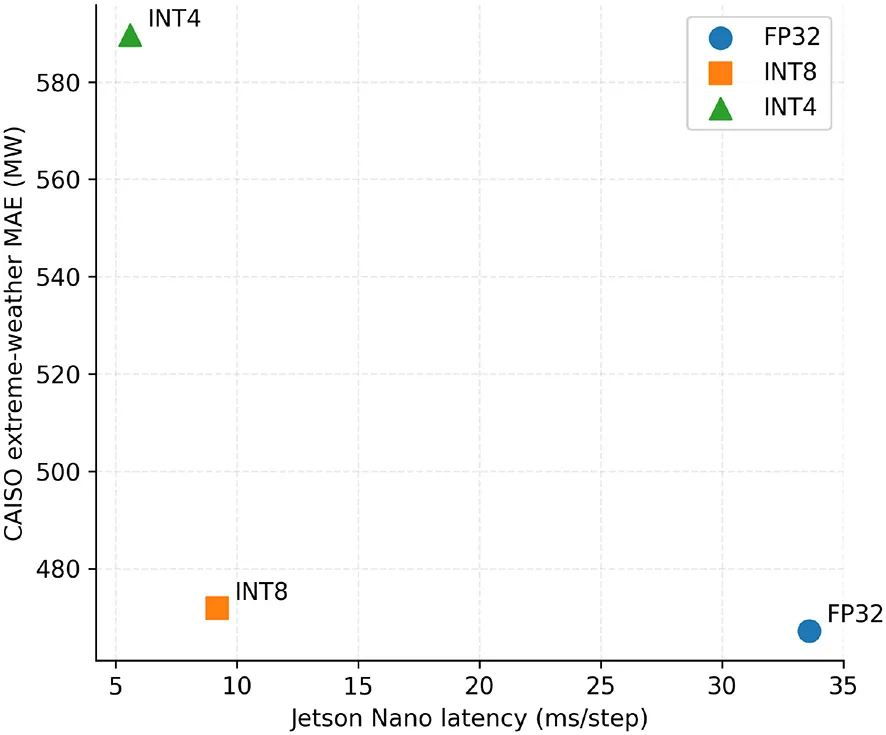
Quantization ablation: extreme-weather MAE on the CAISO test partition vs. Jetson Nano inference latency for three precision levels. Each point shows the mean over five runs; error bars show ±1 standard deviation. FP32 achieves 467.2 MW at 33.6 ms. INT8 achieves 471.8 MW at 9.2 ms, a 3.65 × speedup with a 0.98% relative MAE increase. INT4 achieves 589.7 MW at 5.6 ms, a 6.00 × speedup but with a 26.2% relative MAE increase. The INT8 operating point circled in the figure is the Pareto-optimal choice for this application.

### 6.5 Qualitative demand response analysis

Table 9 shows three representative DR Decision Engine outputs from the CAISO extreme-weather test partition, illustrating the scaling of the alert severity to the predicted exceedance magnitude.

**Table 9:** Representative output examples from the DR Decision Engine on the CAISO extreme-weather test partition.

Weather context	6-Step forecast (MW)	δ (MW)	DR action
Temp: 44.1 °C, Wind: 6.2 m/s, *f*_ext_ = 1	[46,000, 48,200, 49,500, 50,100, 49,700, 49,000]	+2, 100	ACTIVATE_ DEMAND_ CURTAILMENT (WARNING)
Temp: 36.8 °C, Wind: 4.1 m/s, *f*_ext_ = 1	[45,900, 47,100, 48,100, 48,500, 48,100, 47,400]	+500	ISSUE_ CONSERVATION_ NOTICE (ADVISORY)
Temp: 24.3 °C, Wind: 2.8 m/s, *f*_ext_ = 0	[40,700, 40,200, 39,600, 39,000, 38,600, 38,200]	−7, 300	RESTORE_ DEFERRED_ LOAD (RESTORE)

Each row corresponds to one inference call. The 6-step forecast shows the CE-LSTM's predicted grid load trajectory; δ is the exceedance above the operator-configured threshold of *P*_th_ = 48, 000 MW; and the DR action column shows the structured recommendation dispatched to the operator.

### 6.6 Discussion

Several findings merit discussion beyond the primary accuracy metrics.

The binary extreme-weather flag *f*_ext_ accounts for a 31.7% MAE reduction when present in the CAISO evaluation, despite being a zero-computation-cost indicator derived from simple threshold rules on already-available weather data. This suggests that a large share of the achievable improvement in extreme-weather forecasting may come from explicit event encoding rather than complex meteorological feature engineering. This finding aligns with the load forecasting study by Lu et al. (2026), which identified that models overfit to normal conditions and underestimate demand peaks because they fail to capture the non-stationary shifts induced by extreme events.

The consistent improvements across CAISO (summer heatwave, West Coast) and ISONE (winter cold snap, East Coast) provide evidence of transferability across two contrasting climate regimes without dataset-specific architectural modifications. While two case studies provide limited evidence for generalization across climate regimes, the results indicate that the CE-LSTM methodology transfers between these two specific, contrasting conditions. The WSI formulation adapts to each regime through the sign of the temperature term (Equations 1, 2), while all other hyperparameters and the training procedure remain identical, which supports, but does not by itself establish, scalability to additional grid operators and climate zones.

The DR Decision Engine, by design, uses rule-based severity tiers rather than a learned DR policy. This is an intentional design choice: a learned policy would require reinforcement learning from historical operator decisions or imitation learning from dispatch logs, which constitutes a separate research contribution and requires data not available in the open domain. The rule-based engine provides a structured, interpretable interface between the probabilistic forecast and the operator, ensuring that DR recommendations are predictable and auditable. Integration with a learned policy is identified as a future direction in Section 7.

### 6.7 Limitations

Several limitations should be acknowledged. First, both datasets cover limited time periods (28 months for CAISO, 8 months for ISONE). While these periods include documented extreme-weather events of operational significance, the extreme-weather partitions, particularly for ISONE at 58 test records, are modest in size. Bootstrap confidence intervals over 1,000 resamples of the CAISO extreme-weather test partition produce a 95% CI for CE-LSTM MAE of [426.8, 487.7] MW, confirming that the estimate is stable. For ISONE, the 95% bootstrap CI is [263.5, 362.1] MW, a wider relative interval reflecting the smaller test partition. Second, the CAISO validation split used for early stopping and hyperparameter selection contains only 18 extreme-weather records (0.6% of the split); while the bootstrap CI above suggests the final test-set MAE estimate is stable, future work should confirm that the model-selection procedure itself is insensitive to this small validation subset by comparing checkpoints selected via extreme-weather MAE against those selected by full-set validation loss. Third, the ISONE training partition contains only 8 extreme-weather records out of 4,069 (0.2%), since it covers predominantly the July–December 2023 period before the coldest weather arrives (Table 3); the model has minimal labeled exposure to cold-snap conditions during ISONE training, and its extreme-weather performance on that dataset derives from the continuous Weather Severity Index and the weather-weighted loss rather than from the binary extreme-weather flag, which is uncorrelated with load in the ISONE training partition (*r* = 0.01, Section 4.2). Fourth, hyperparameters (learning rate, batch size, dropout) were tuned once via grid search and shared across all deep-learning baselines rather than tuned per architecture (Section 5.1); this may disadvantage distinct models such as the iTransformer relative to what tuned hyperparameters could achieve, and the reported baseline comparisons should be interpreted with this shared-tuning limitation in mind. Fifth, both datasets derive meteorological inputs from a single geographic reference point per grid operator. Distribution-level analysis would require spatially resolved weather data matched to individual feeder locations. Sixth, the DR Decision Engine uses a fixed operator-specified capacity threshold *P*_th_; dynamic thresholds sourced from a state estimator would provide greater operational value but require real-time SCADA integration not available in the open domain. Seventh, the results on two US grid operators may not generalize to grids with different demand profiles, such as European interconnected systems with higher renewable penetration or South Asian systems with monsoon-driven load patterns.

## 7 Conclusion

This paper presented ClimateEdge, an end-to-end framework for real-time demand response in smart grids under extreme weather conditions, evaluated on two independent grid datasets spanning contrasting climate scenarios. Three core contributions were demonstrated: a weather-augmented feature pipeline incorporating a Weather Severity Index and an extreme-weather binary flag; the CE-LSTM, combining stacked LSTM layers, temporal attention, and a weather-weighted loss function; and an INT8 quantization and DR decision pipeline operational on a Jetson Nano at 9.2 ms per inference step with 8.1 MB runtime footprint.

On the CAISO summer heatwave partition the CE-LSTM achieves a MAE of 471.8 MW and MAPE of 1.55%, outperforming the iTransformer by 21.1% and the Vanilla LSTM by 44.9%. On the ISONE winter cold-snap partition it achieves 329.6 MW MAE and 2.31% MAPE, outperforming the iTransformer by 19.4%. The INT8 operating point delivers a 3.65 × latency speedup at less than 1% accuracy degradation, and the weather feature ablation confirms that the binary extreme-weather flag alone accounts for a 31.7% MAE improvement. The consistency of results across two distinct grid operators provides evidence of transferability between these two contrasting regimes, though generalization claims would require evaluation on additional grid operators.

Future work will focus on four directions. First, validation on additional grid operators, including systems with high renewable penetration, to evaluate transferability beyond the two US ISO markets studied here, as this is the most important step toward establishing broader applicability of the framework. Second, integration of numerical weather prediction forecast data as an additional input to address the onset-lag errors identified in the qualitative analysis. Third, investigation of learned DR policy components, using imitation learning from historical operator dispatch decisions, as a complement to the current rule-based engine. Fourth, given the framework's emphasis on extreme conditions and edge operation, a comparison against Hierarchical Temporal Memory (HTM) (Román-Portabales and López-Nores, 2026) as an alternative forecasting paradigm may be valuable for studying continual on-device adaptation to weather-driven distribution shifts, a capability the current batch-trained CE-LSTM does not provide.

## Data Availability

Grid load data is publicly available from the CAISO and ISONE grid operators via the gridstatus Python library (https://github.com/gridstatus/gridstatus). Meteorological data is available from the Open-Meteo historical weather archive (https://open-meteo.com).
